# Cluster equilibrium scheduling method based on backpressure flow control in railway power supply systems

**DOI:** 10.1371/journal.pone.0243543

**Published:** 2020-12-09

**Authors:** Zhijian Qu, Hanxin Liu, Hanlin Wang, Xinqiang Chen, Rui Chi, Zixiao Wang

**Affiliations:** 1 Electrical and Automation Engineering College, East China Jiaotong University, Nanchang, Jiangxi, China; 2 Institute of Logistics Science and Engineering, Shanghai Maritime University, Shanghai, China; University of Birmingham, UNITED KINGDOM

## Abstract

The purpose of the study is to solve problems, i.e., increasingly significant processing delay of massive monitoring data and imbalanced tasks in the scheduling and monitoring center for a railway network. To tackle these problems, a method by using a smooth weighted round-robin scheduling based on backpressure flow control (BF-SWRR) is proposed. The method is developed based on a model for message queues and real-time streaming computing. By using telemetry data flow as input data sources, the fields of data sources are segmented into different sets by using a distributed model of stream computing parallel processing. Moreover, the round-robin (RR) scheduling method for the distributed server is improved. The parallelism, memory occupancy, and system delay are tested by taking a high-speed train section of a certain line as an example. The result showed that the BF-SWRR method for clusters can control the delay to within 1 s. When the parallelism of distributed clusters is set to 8, occupancy rates of the CPU and memory can be decreased by about 15%. In this way, the overall load of the cluster during stream computing is more balanced.

## Introduction

In recent years, as modern high-speed railway technology rapidly develops, power supply systems and equipment for high-speed railways have been key to effective railway transportation. Correspondingly, increasingly more types of information are collected: not only isolating switches of overhead contact system (OCS) but also communication, ring main unit, signals, and 10/0.4 kV low voltage substations were monitored [1–3]. As a result, the data acquired by the railway power supply monitoring system exponentially increased [4]. They amount to more than 50 times that obtained through the monitoring system for normal and fast-speed railways, generating big data in the railway power supply monitoring system. In the presence of massive data information, how to screen alarm messages rapidly has become the difficulty in, and focus of the rapid response technology implementation with massive information pertaining to a railway power supply [5]. Therefore, it is necessary to explore the technical scheme with low delay and high utilization of clusters for solving massive alarm messages on railway power supply using related techniques of big data analysis [6, 7].

A railway power supply system is a special industrial power transformation and distribution network system [8, 9]. When faced with data at the 10^6^ level, relational databases suffer from slow response times and poor scalability. New fields can only be extended through association listing, which causes data to be increasingly redundant. Therefore, cluster processing technology is generally applied to deal with massive power data. At present, the cluster processing technology for big data of power is mainly divided into three types [10, 11]: batch processing technology by taking MapReduce as the core [12]; data processing technology based on the main memory database; and the model for big data stream computing based on memory [13]. In terms of batch processing technology, the data transmitted from an uplink are subjected to isochronal treatment by utilizing MapReduce technology; however, it is necessary to acquire data from disks and then transmit the intermediate result back to the disks which results in a high I/O (input/output) overhead. Additionally, if message queues are irregularly input into a data processing system, isochronal intervals [14] can lead to message accumulation. Therefore, the batch processing technology is inapplicable when dealing with alarm messages with a high requirement for timeliness [15]. As for that data processing technology based on the main memory database, all telemetry data are always cached to memory. The system delay is 10 to 1000 times lower while throughputs are 100 to 1000 times larger compared with the data processing based on disks, however, the technology exhibits volatile memory so that it is necessary to copy and back-up data to disks. Thus, the method incurs high energy consumption, being 50 to 100 times that of a disk system, and costs are high for data applications occupying a large amount of memory. Additionally, oversize memory occupancy leads to the decrease of the efficiency of data retrieval and access. Therefore, the technology is unsuitable for railway power supply centers with large data size and having a high requirement for access efficiency. In terms of the model for big data stream computing based on memory [13, 14, 16, 17], each record of big data on railway power is converted to an n-tuple-stream data Spout through stream computing.

In [18], Wrangler is a system that actively avoids situations that lead to laggards. Wrangler automatically predicts such situations using statistical learning techniques based on cluster resource utilization. However, when processing task scheduling, the cluster resource consumption will be significantly improved, which is not applicable to the simultaneous arrival of massive data in the railway power supply cluster center [19, 20].

In [21], the traditional flow calculation is adjusted to handle micro-batch records, thus dividing the infinite stream into finite blocks which can provide good performance in terms of large-scale data fault tolerance. Due to the inherently equal interval characteristics of micro-batch processing, if the execution time of the task exceeds the interval time, it will however not only affect the delay of the current micro-batch but also affect the subsequent queuing delay, which gives rise to serious backwardness problems.

In [22], a particle swarm optimization (PSO) algorithm is used to solve optimization problems in task scheduling. It is based on swarm intelligence to find approximate solutions, but when using the PSO algorithm to schedule a large number of tasks, it will take up too much computer resource and reduce the responsiveness of the system.

The polling scheduling algorithm is also a common strategy for task balancing scheduling. Generally, the CPU scheduler loops through the entire ready queue, and allocates no more than one time slice CPU for each process: however, this method does not consider the actual processing performance difference of the server, resulting in an imbalance between nodes.

According to the existing processing methods of the railway power supply monitoring system and the resource problem of information transmission [23], cluster resource utilization and system delay are used as the main indicators to evaluate the algorithm. The improved RR algorithm in the data center is a time slice-based scheduling method, which can control the delay within 1s and weight the RR algorithm. The goal is to differentiate the servers in the data center the better to balance the cluster nodes. For the problem of data processing timeout failure in a burst-rich data stream, it is best to set a buffer at the data source, and then trigger the backpressure mechanism after the buffer is exceeded. The purpose of this is to ensure load-balancing in the system, so as to achieve more flexible data flow control [24–26]. This research improves the polling scheduling algorithm, a smooth weighted round-robin scheduling method based on back-pressure and flow control (BF-SWRR) is proposed and a model for multi-node stream computing is established. Based on engineering examples, the response time of data and occupancy of resources in servers of the model can be validated.

This paper focuses on the application of a backpressure flow control mechanism and balanced polling scheduling in the railway power supply big data monitoring center. In the second section, the railway distributed intelligent power supply network system model and its processing flow are proposed. In the third section, a queuing model for railway power distribution monitoring cluster information based on backpressure flow control is constructed to alleviate the pressure on the information dispatching center under conditions involving information blowout, thus avoiding the loss of key data information. In the fourth section, the server weights in the cluster are reasonably allocated, and the polling scheduling algorithm is improved to make full use of the idle node resources. In the fifth section, the data of the power distribution station of a certain local train are taken as an actual engineering example. It is verified that the proposed method can balance the resource scheduling between nodes and reduce the delay in processing data.

## Stream computing process for massive data from a railway power distribution network

**Fig 1:**
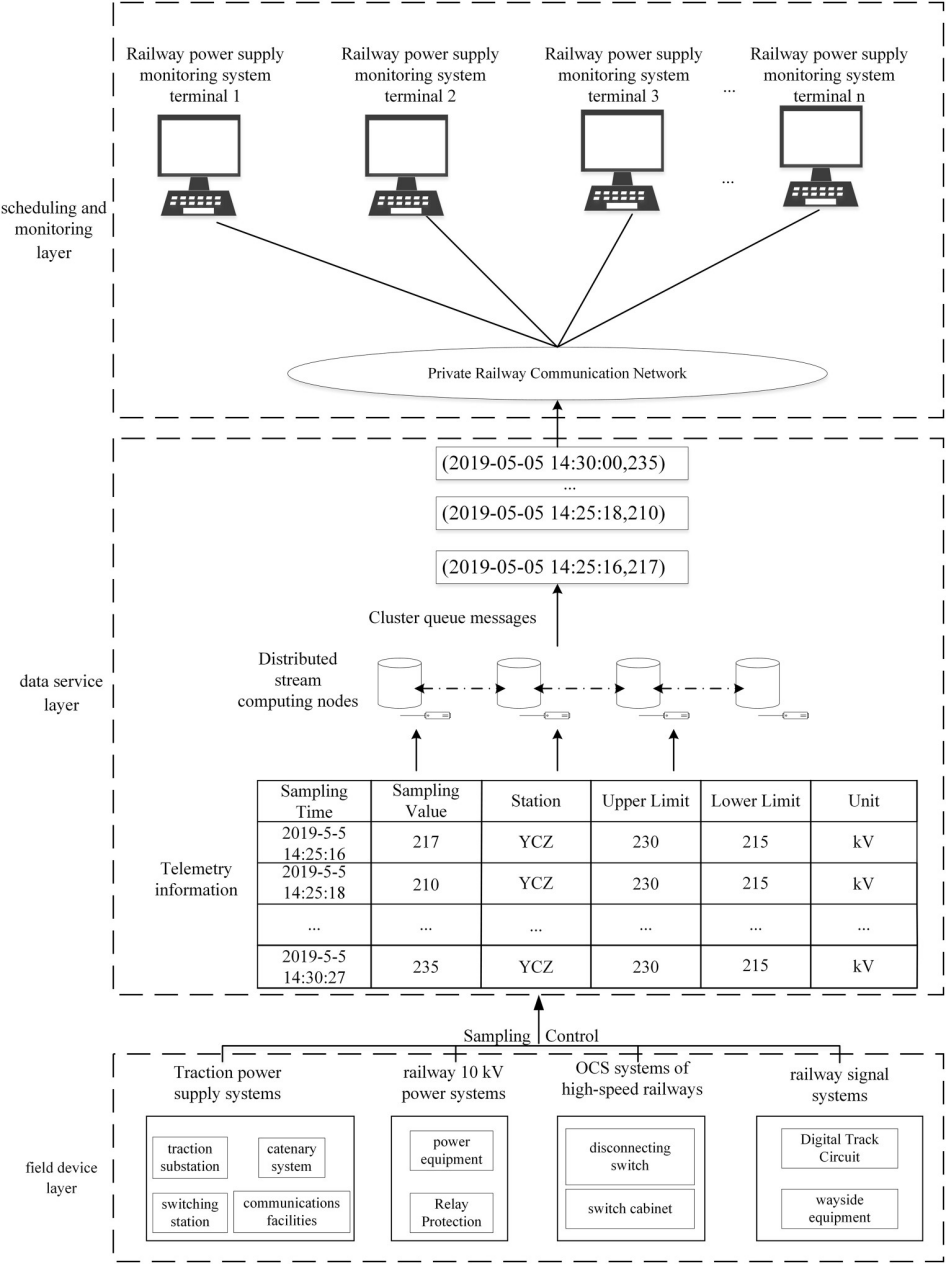
Cluster monitoring and processing model for data from high-speed railway power supply system. (The figure is similar but not identical to the original image and is therefore for illustrative purposes only).

An intelligent distributed power supply network system for railways is developed rapidly. Sensors are installed in communication devices, wayside equipment, OCS, and auxiliary equipment along railways for monitoring voltage, current, temperature, humidity, and wear loss of wire rods. Moreover, the sampling frequency also increasingly grows with the construction of high-speed railways, thus generating massive system data [27]. The specific structures are shown in Fig 1.

The monitoring and processing model for massive data from the power supply system of high-speed railways mainly consists of a scheduling and monitoring layer, a data service layer, and a field device layer. The field device layer is mainly composed of sensors used for traction power supply systems, 10 kV railway power systems, OCS systems of high-speed railways, and railway signal systems. The telemetry data are uploaded to the data service layer [28]. Owing to data volumes being large and time being non-uniformly distributed, data overload often occurs in severe cases, which cannot be solved by using traditional databases. Therefore, distributed stream computing is employed: the initial data processing is carried out in the memory of distributed servers to reduce the I/O access time thereof, thus improving the throughput and processing speed of data. The data transmitted from a private railway communication network are recalled to the human-computer interaction interface through the scheduling and monitoring layers [29]. By doing so, the abnormal conditions of field devices can be found timeously to reduce the probable stagnation of the interface screen caused by small volume limits on traditional databases.

We collected the actual data obtained based on measuring points in a railway EMU (including SampleOrder, DescName, RTUNo, and ID). The telemetry data are taken as the data source for stream processing. The components of the stream computing topology programming model mainly include Spout and Bolt. The Spout is the source of monitoring topology flows, typically reading data from external data sources (SCADA data tables) and then converting them to source data within the topology. The Bolt is a link to data processing in topology. Bolt can monitor the sensor monitoring voltages, currents, temperatures, humidities, and wire wear of an important power distribution station, and transmit the results to the interface of the monitoring system and store them in the database. The stream computing model can cut fields in the telemetry information table to acquire a data-tuple stream, which is constantly uploaded to the monitoring layer to find over-limit messages timeously.

## A queueing model for monitored cluster information about railway power distribution based on backpressure flow control (BF)

**Fig 2:**
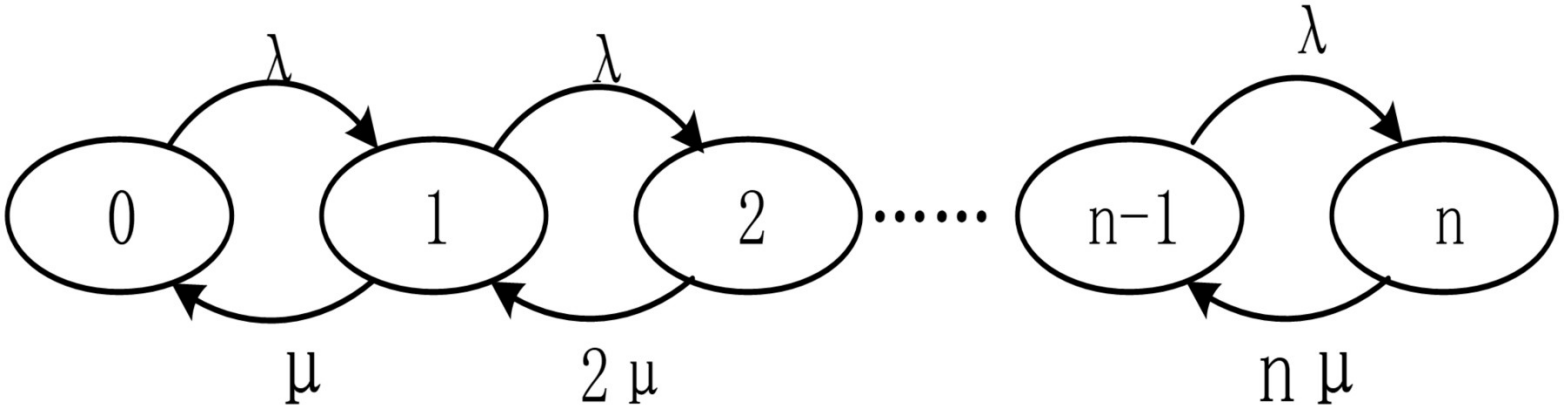
State transition of message queues in the monitoring center.

When transmitting communication data to the scheduling center, the following three conditions are satisfied [30]:

1. Communication service shows stochasticity and the communication traffic available per unit time is mutually independent of the time at the message service.
2. On condition of having sufficiently low Δ*t*, within the interval [*t*, *t*+Δ*t*), the probability that one message reaches the scheduling center is independent of time while is positively proportional to the interval length, that is,
  $$P_{1}(t,t+\Delta t)\;=\;\lambda \Delta t+O(\Delta t)$$ (1)
  where, in the case of Δt → 0, O(Δt) represents the infinitesimal of higher-order about Δt; λ > 0 is a constant, λ is the intensity of the probability.
3. For sufficiently low Δt, within the time interval [*t*, *t*+Δt), the probability that two or more messages reach the scheduling center is so small that can be ignored, that is:
  $$\sum_{n\;=\;2}^{\infty }P_{n}(t,t+\Delta t)\;=\;O(\Delta t)$$ (2)
  Therefore, the probability that data are transmitted back to the scheduling center conforms to the Poisson distribution, with the arrival rate λ. Generally, the service time of servers in the scheduling and monitoring system conforms to a negative exponential distribution, so the function of time of servers for processing data is expressed as follows:
  $$P(v\leq t)\;=\;1-e^{-\mu t}\;(t\geq 0)$$ (3)
  where, *μ* denotes the work efficiency of a single server node; thus, 1/*μ* represents the time taken by a single server node in processing data; it is supposed that $\rho_{1}\;=\;\frac{\lambda }{\mu },\rho \;=\;\frac{\lambda }{n\mu }$. Therefore, the model can be expressed as M/M/n, that is, the inflow process of messages is a Poisson flow; the time taken by server nodes to process data conforms to a negative exponential distribution and there are *n* servers in the scheduling and monitoring center. It is supposed that the information queue has infinite volume, so the possible state set of the queue is displayed as Φ = {0, 1, 2, …}. Therefore, the state transition of the message queues is as presented in Fig 2.

In Fig 2, the state k (0 ≤ k ≤ n) represents the fact that *k* server nodes within the scheduling center are processing an information flow *in situ* while the other *n*-*k* server nodes are idle; with *k* (*k* > *n*) server nodes, *n* server nodes are occupied with processing information flow while other server nodes are queued for data processing. It is assumed that there is only a waiting queue at present; on this condition, when there are idle server nodes, information flow waiting in the queue is successively processed. In the case that the system is in equilibrium, the expected value of the average queue length of information flow is L_q_; the expected value of average waiting time for processing information flow is T_q_; the expected value of the average processing time of information flow is T_s_. The probability that the number of messages in queues is *n* is calculated as P_n_. Therefore, K–algebraic equations can be attained:

Pk={ρ1kk!p0=nkk!ρkp0'(0≤k<n)ρ1kn!nk-np0=nkk!ρkp0'(k≥n) (4)

According to the regularity condition $\sum_{k\;=\;0}^{\infty }p_{k}\;=\;1$, on condition of ρ < 1, the following equations can be obtained:

$$(\sum_{k\;=\;0}^{n-1}\frac{\rho_{1}^{k}}{k!}+\sum_{k\;=\;n}^{\infty }\frac{\rho_{1}^{k}}{n!n^{k-n}})p_{0}$$

$$=\;(\sum_{k\;=\;0}^{n-1}\frac{\rho_{1}^{k}}{k!}+\sum_{k\;=\;n}^{\infty }\frac{\rho_{1}^{n}}{n!(1-\rho )})p_{0}\;=\;1$$ (5)

thus,

$$p_{0}\;=\;{\left(\sum_{k\;=\;0}^{n-1}\frac{\rho_{1}^{k}}{k!}+\sum_{k\;=\;n}^{\infty }\frac{\rho_{1}^{n}}{n!(1-\rho )}\right)}^{-1}$$ (6)

According to Little’s law, when the information flow to the scheduling and monitoring center delivered from the field is stable, the average queue length L_q_ and work queue length L_S_ in the scheduling center in the system at any time are expressed as follows:

$$L_{q}\;=\;\sum_{k-n}^{\infty }(k-n)p_{k}\;=\;\sum_{l\;=\;1}^{\infty }lp_{l+n}$$

$$=\;\frac{\rho {\left(n\rho \right)}^{n}}{n!}p_{0}\sum_{l\;=\;1}^{\infty }l\rho^{l-1}$$

$$=\;\frac{\rho_{1}^{n+1}}{(n-1)!{\left(n-\rho_{1}\right)}^{2}}p_{0}$$ (7)

$$L_{s}\;=\;L_{q}+\rho_{1}$$ (8)

The average waiting time W_q_ and the job-processing time W_s_ for the information flow of the controlling center are demonstrated as follows:

$$W_{q}\;=\;\frac{L_{q}}{\lambda }\;=\;\frac{\rho_{1}^{n}p_{0}}{\mu n∙n!{\left(1-p\right)}^{2}}$$

$$\;=\;\frac{{\left(\frac{\lambda }{\mu }\right)}^{n}{\left(\sum_{k\;=\;0}^{n-1}\frac{\rho_{1}^{k}}{k!}+\sum_{k\;=\;n}^{\infty }\frac{\rho_{1}^{n}}{n!(1-\rho )}\right)}^{-1}}{\mu n∙n!{∙(1-\frac{\lambda }{n\mu })}^{2}}$$ (9)

$${\;W}_{s}\;=\;\frac{L_{s}}{\lambda }\;=\;W_{q}+\frac{1}{\mu }$$ (10)

By analyzing the above equations, it can be seen that, when the information flow is steadily transmitted, the work efficiency of nodes exerts a significant influence on the processing time W_s_ and waiting time W_q_. It is necessary to assign reasonable weights to each node and schedule server nodes, thus greatly reducing the processing time W_s_; however, in the case of having an excessively large arrival rate λ, server nodes crash under information overload. Especially when the server nodes allocated for scheduling cannot withstand the pressure exerted by massive data, resulting in a scheduling failure, various nodes are needed for data service crash.

**Fig 3:**
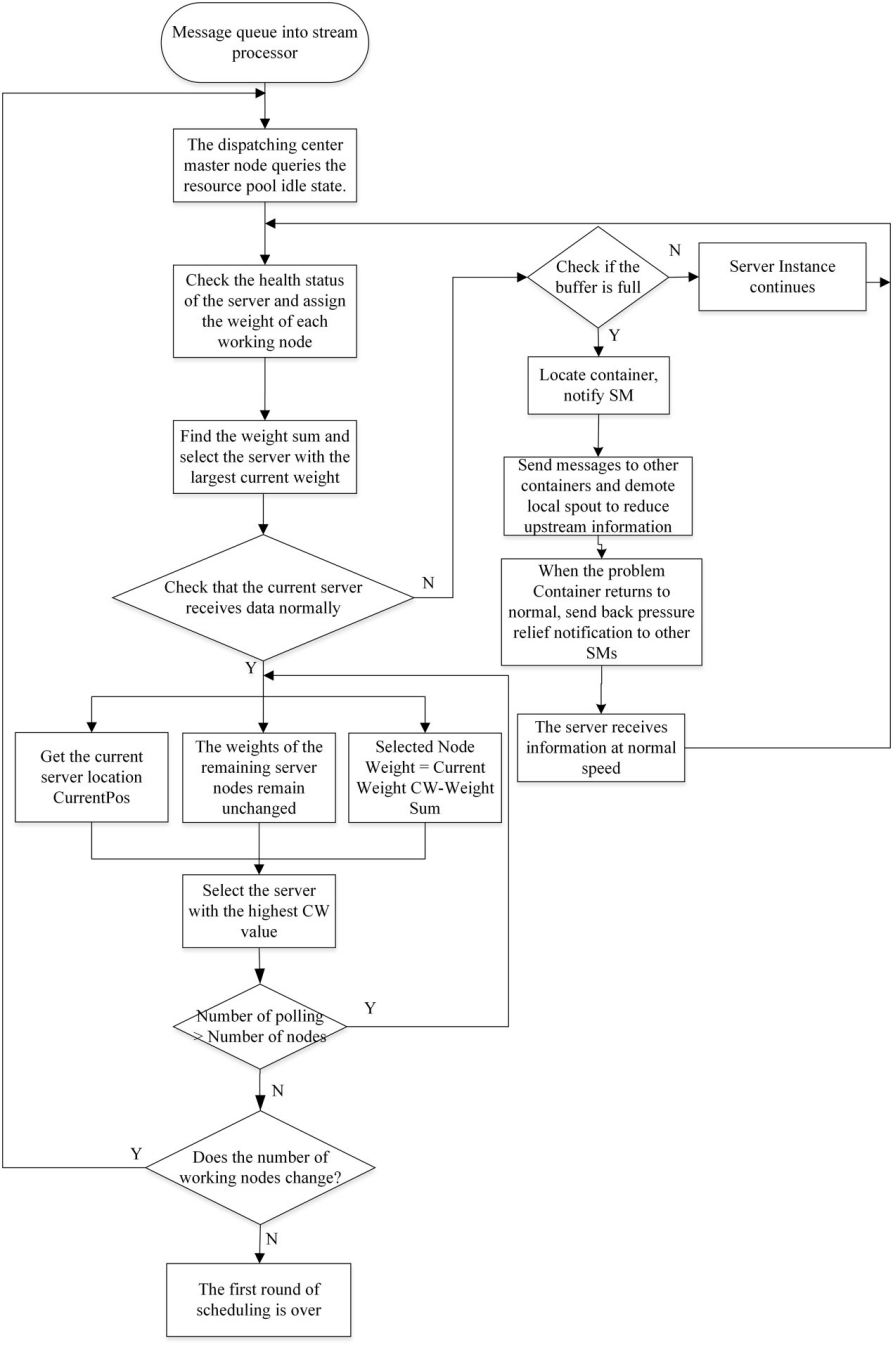
Data processing at server nodes.

To prevent the aforementioned situation, a backpressure mechanism is introduced to the front end of the stream processor. In the cluster monitoring center for railway scheduling, the arrival of data shows certain time regularity [31, 32]. From 06:00–24:00, high-speed and normal-speed railways are all busy, with large data sets generated. In this case, data overload likely appears and the data flow from a telemetry station can reach 90 groups per second. Moreover, a scheduling and monitoring center generally needs to control hundreds of telemetry stations so that dataset sizes can reach a magnitude of 10^4^ within one second. At the onset of data overload, it is possible that alarm data fail to be uploaded to the scheduling center when time delay lasts for 10 s, which is likely to lead to a late report, omission, or false report of crucial alarm messages. Therefore, it is necessary to set a certain data threshold: when reaching the threshold after being processed by nodes, timeous back-pressure treatment is required to separate the data, avoiding delay in data processing (Fig 3).

### Cluster scheduling mode in stream computing for the monitoring center of a railway system

#### Weight assignment of weighted RR scheduling

The big data system for railway power supply is generally composed of multiple nodes, each of which comprises different resources. In general cases, the condition is considered as a RR model to be analyzed and improved [18].

The basic model for the RR system consists of a master node and *n* slave nodes. According to a certain rule, the master node successively operates each slave node in the queue in a given direction and the first slave node repeatedly runs after the last slave node in the queue completes this operation. In this way, multiple nodes share a resource pool, and the nodes having service demand possess the right to utilize resources.

Therefore, how to assign reasonable weights to servers is the key to improving the algorithm.

At first, memory, CPU, and disk space are determined as the indices for evaluating the processing capacity of servers for stream computing. By taking a computing task of screening topologies of telemetry information as an example, if task instance I_i_ is distributed to a container C_j_ of node n_k_, the resources of CPU, memory, and disk required by each instance are C_is_, M_is_, and D_is_, respectively; each container needs the resource of C_cs_, M_cs_, and D_cs_ for CPU, memory, and disk, respectively; the resources of CPU, memory, and disk required by each node are C_n_, M_n_, and D_n_, respectively. Each node contains one or multiple containers. The constraint conditions on resources are as follows:

$$\sum C_{is}+\sum C_{cs}\leq \;\propto C_{n}$$ (11)

$$\sum M_{is}+\sum M_{cs}\leq \;\beta M_{n}$$ (12)

$$\sum D_{is}+\sum D_{cs}\leq \;\gamma D_{n}$$ (13)

In a big-data monitoring system for railway power supply, to prevent each worker node from being at full load, which leads to system stagnation and non-smooth scheduling, it is necessary to reserve a certain resource for each worker node. Therefore, the above α, β, and γ are separately threshold parameters of resources set by dispatchers and the parameters can be adjusted according to requirement.

Generally, a cluster contains *n* evaluation indices, whose weighting coefficients are determined according to m server nodes, thus forming an original data matrix X (occupancy rates of CPU and memory, and disk resource). $X_{ij}\;=\;{\left(x_{ij}\right)}_{n\times m},(i\;=\;1,2,\ldots ,n;j\;=\;1,2,\ldots ,m)$ and containers distributed for nodes generally follow a certain standard and occupy few resources.

1. The occupancy rate C_is_ of CPU, occupancy rate M_is_ of memory, and disk resource D_is_ of a task instance are subjected to data standardized by the entropy method. The standardized matrix is expressed as R:
  $$R\;=\;[\begin{array}{ccc} R_{11} & \cdots & R_{1j}\\ \vdots & \ddots & \vdots \\ R_{i1} & \cdots & R_{ij} \end{array}](R_{ij}\;=\;\frac{x_{ij}-min\;(x_{ij})}{\text{max}\;\left(x_{ij}\right)-min\;(x_{ij})})\;$$ (14)
2. Definition of entropy: it is supposed that there are *n* evaluation indices and *m* server nodes, the initial data matrix is *X* = {*r_ij_*}_*m*×*n*_ and *f_ij_* represents the proportion of the index value of the *j*^th^ evaluated object in the *i*^th^ index. So the entropy *H*_*i*_ of the *i*^th^ evaluation index is defined as follows:
  $$f_{ij}=\frac{r_{ij}}{\sum_{i=1}^{n}r_{ij}}(i=1,2,\ldots ,n;j=1,2,\ldots ,m)$$ (15)
  $$k=\frac{1}{\text{ln}m}$$ (16)
  $$H_{i}=\frac{1}{\text{ln}m}\sum_{j=1}^{m}f_{ij}\text{ln}\frac{1}{f_{ij}}=-k\sum_{j=1}^{m}f_{ij}\text{ln}f_{ij}$$ (17)
3. Calculation of discrepancy coefficient g_j_, the ratio D of discrepancy coefficients to mapping value R is:
  $$g_{i}=1-H_{i}$$ (18)
  $$D=\frac{\text{max}(1-H_{i})}{\text{min}(1-H_{i})}$$ (19)
  $$R=\sqrt[{a-1}]{\frac{D}{a}}$$ (20)
  where a refers to dynamic adjustment coefficient; *a* is equivalent to the integer which is most approximate to *D* on condition of D ≤ 9; *a* = 9 in the case of *D* > 9. The purpose of calculating the (a-1)th root of *D/a* is to calculate the mapping value of *D* evenly distributed on 1 to 9 scales.
4. The mapping values of 1 to 9 scales are obtained by calculating the power (corresponding scale minus 1) of the R scaling value to multiply by 1 to 9 scales. The corresponding relationships between 1 to 9 scales and their scaling values are listed in Table 1.
5. Establishment of a judgment matrix
  The ratio $r_{jk}\;=\;\frac{g_{j}}{g_{k}}$ of discrepancy coefficients between each pair of evaluation indices is calculated. When $r_{jk}\;=\;\frac{g_{k}}{g_{j}}$ is calculated. By taking the scale corresponding to the minimal difference between r and mapping value of scale as the comparison result, if r_jk_ approximates to the mapping value 6R^5^, the relative importance between indices j and k is 6.
6. After establishing the judgment matrix based on information entropy, the weighting method is the same as AHP in calculating the weights of indices. Moreover, owing to the relative importance being attained according to the ratio of discrepancy coefficients, it is unnecessary to conduct a consistency check.
  The weighting coefficients of various indices are expressed as follows:
  $$W_{i}=\frac{g_{i}}{\sum g_{i}}=\frac{1-H_{i}}{\sum 1-H_{i}}=\frac{1+k\sum_{j=1}^{m}f_{ij}\text{ln}f_{ij}}{\sum 1+k\sum_{j=1}^{m}f_{ij}\text{ln}f_{ij}}$$ (21)

**Table 1:** Mapping value of scale.

Scale	1	2	3	4	5	6	7	8	9
**Mapping value**	1*R*^0^	2*R*^1^	3*R*^2^	4*R*^3^	5*R*^4^	6*R*^5^	7*R*^6^	8*R*^7^	9*R*^8^

However, in practice, the request can cause nodes with a high weight to be frequently invoked, so excessive tasks are assigned, and the computer may crash in severe cases, therefore, the weighted RR scheduling method is improved by introducing smooth weighted round-robin (SWRR) scheduling.

#### SWRR scheduling method for comprehensively monitoring railway power supply

It is supposed that a cluster contains *n* server nodes S. According to improved entropy method, the distributed and effective weights are calculated as W and Current_WeightSum (CW) = {cw_1_, cw_2_, cw_3_, …, cw_n_}.

Apart from including a distributed weight, each server also has a currently effective weight cw_i_, whose initial value is cw_0_. The indicator variable CurrentPos represents the ID of the currently selected server with an initial value of -1. The sum of weights of all servers remains unchanged. The scheduling algorithm can be described as follows:

Step 1: (1) The currently effective weight CW_i_ of each instance i is equivalent to the distributed weight W_i_ in the initial stage;
     (2) The sum weightSum of distributed weights is calculated;
Step 2: (1) The instance with the maximum currently effective weight is selected;
     (2) The sum weightSum of weights of all instances is subtracted from CW_i_ of the selected instance and variable currentPos point to the instance;
Step 3: (1) The distributed weight W_i_ is added to the currently effective weight CW_i_ of each instance i;
     (2) All servers are sorted in descending order according to the weights;
Step 4: The instance, to which the variable currentPos points is selected as the working server;
Step 5: Steps 2, 3, and 4 are repeated during each scheduling operation.

For example, it is supposed that there are four server nodes in the big data monitoring cluster of a railway power distribution network, with distributed weights of {5,1,1,2}. The scheduling mode is summarized in Table 2.

**Table 2:** Scheduling request of SWRR.

Request	Cw before selecting server	CurrentPos	Selected server	Cw after selecting server
1	(5, 1, 1, 2)	0	A	(-4, 1, 1, 2)
2	(1, 2, 2, 4)	3	D	(1, 2, 2, -5)
3	(6, 3, 3, -3)	0	A	(-3, 3, 3, -3)
4	(2, 4, 4, -1)	1	B	(2, -5, 4, -1)
5	(7, -4, 5, 1)	0	A	(-2, -4, 5, 1)
6	(3, -3, 6, 3)	2	C	(3, -3, -3, 3)
7	(8, -2, -2, 5)	0	A	(-1, -2, -2, 5)
8	(4, -1, -1, 7)	3	D	(4, -1, -1, -2)
9	(9, 0, 0, 0)	0	A	(0, 0, 0, 0)

By analyzing Table 2, it can be seen that within a scheduling period, the weights of server nodes in each cluster for solving telemetry information are sorted (in descending order) to obtain corresponding weight sequence CW_1_ = {5, 1, 1, 2}. The server A with the maximum weight is selected to deal with messages; when selecting servers in the second round, the sum of weights is subtracted from the weight of the selected server A in the last round while the weights of the other server nodes remain unchanged, that is, the weight sequence of all server nodes is expressed as CW_2_ = {-4,1,1,2}. The weights of servers are ranked (in descending order) and server C with the maximum current weight CW_2_ is found; in the third round, the same steps are conducted; in the nth (*n* refers to the sum of weights) round, a whole period of scheduling is completed. In this way, the scheduling result is quasi-uniform and the current effective weight returns to {0, 0, 0, 0} in the 9th scheduling stage. In this context, the state of the instance is consistent with the initial state. Therefore, multiple tasks are not repeatedly distributed to the same server node, thus avoiding an accumulation of telemetry data about the railway power supply system.

## BF-SWRR cluster scheduling for a railway power supply system

**Fig 4:**
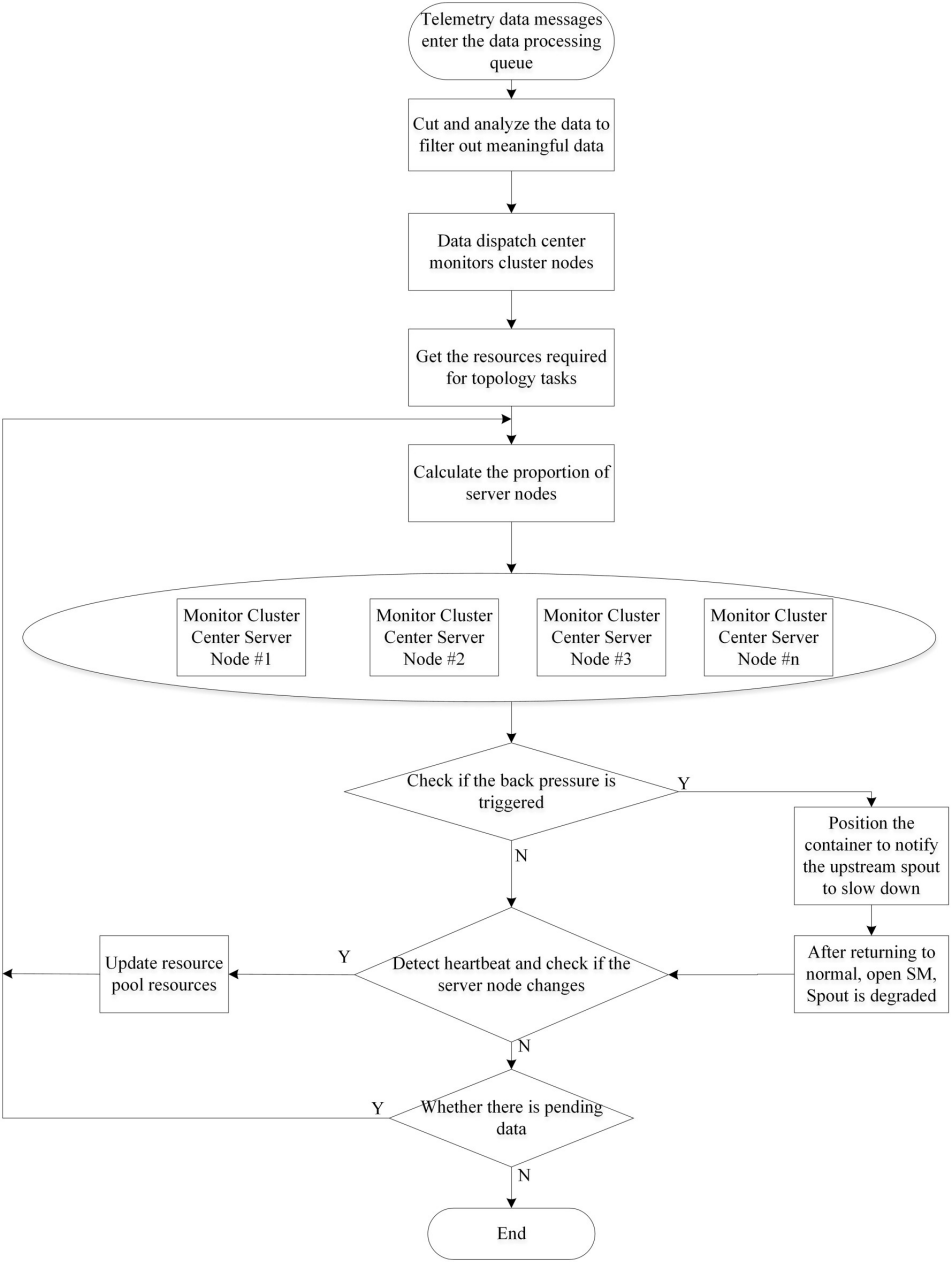
Flowchart for optimization using BF-SWRR scheduling.

As shown in Fig 4, the optimization steps for the scheduling and monitoring center for power supply by using BF-SWRR scheduling are displayed as follows:

Step 1: the actual data (including SampleOrder, DescName, RTUNo, and ID) obtained based on measuring points in situ (Table 3) are taken as the data source for stream processing;
Step 2: the CPU, memory, I/O of the disk of server nodes are all evaluated by the AHP-entropy approach to assigning servers with different weights;
Step 3: according to the state of the servers, the weights applied to server nodes are dynamically adjusted in real time. In this way, the load on each server by utilizing the SWRR scheduling algorithm is not too high so as to prevent reasonable utilization of the current resource pool;
Step 4: according to the characteristics of the information flow model, the information flow is analogous to a Poisson queue model when considering arrival, and service, rates;
Step 5: the backpressure mechanism is introduced to prevent crashes at server nodes caused by the impact of data overload on monitoring clusters of the railway power supply system. The data overload is forcibly degraded through topological treatment and the data flow is controlled at the source to allow processing of data at a stable and reasonable rate.

**Table 3:** Telemetry data.

SampleOrder	DescName	RTUNo	ID	SampleValue	Unit
22	V022	20	0220VBC	14.5	kV
23	V023	20	0220VCA	30.9	kV
24	V024	20	0220I1A	2.5	A

## Case study

**Fig 5:**
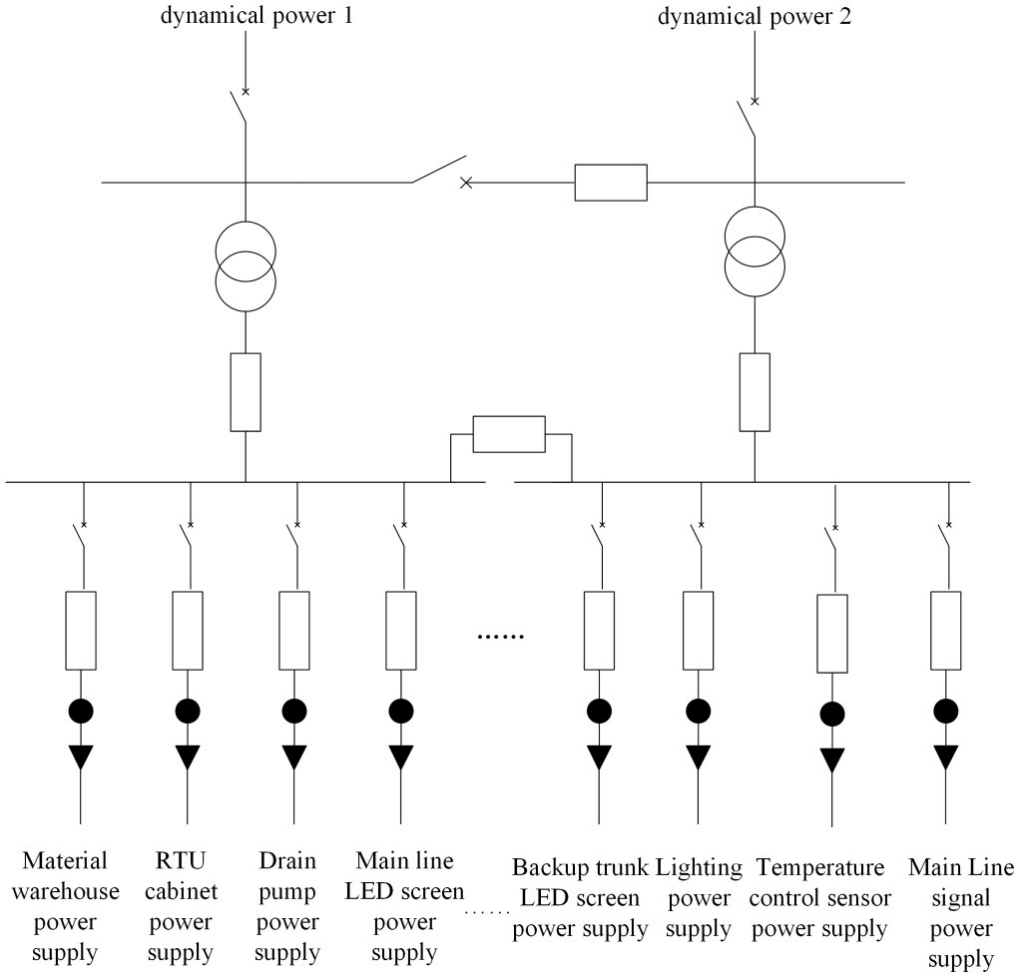
Power supply for a power distribution substation for a high-speed train section.

The measured engineering data in a 10 kV power scheduling and monitoring SCADA system in a certain high-speed train section are taken as a test case (Fig 5).

According to the availability of the two data servers for this power SCADA system and their main backup scheduling servers with redundancy set as in engineering practice, a four-server scheduling and monitoring cluster for a railway power supply system consisting of a master node and three slave nodes is established and its distribution is shown in Table 4.

**Table 4:** The experimental environment of the distributed scheduling and monitoring cluster for railway power supply.

	Computer configuration	OS	Num
**Master node**	CPU: Intel core i5-6500	Ubuntu 16.04 LTS(64 bit)	1
	Dominant frequency: 3.2 GHz		
	Memory: 16 GB		
	Hard disk: 1 TB 7200 rpm		
**Slave nodes**	CPU: Intel core i5-4590		2
	Dominant frequency: 3.3 GHz		
	Memory:8 GB		
	Hard disk: 1 TB 7200 rpm		
	CPU: Intel core i5-6500		1
	Dominant frequency: 3.2 GHz		
	Memory:16 GB		
	Hard disk: 1 TB 7200 rpm		

The telemetry data collected from the monitoring system of the power distribution substation are regarded as the data source, which contains sampling time, the substation ID, the IDs of all monitoring devices, collected voltage, collected current, and temperature recorded by the temperature controller.

### Selection of topological parallelism

**Fig 6:**
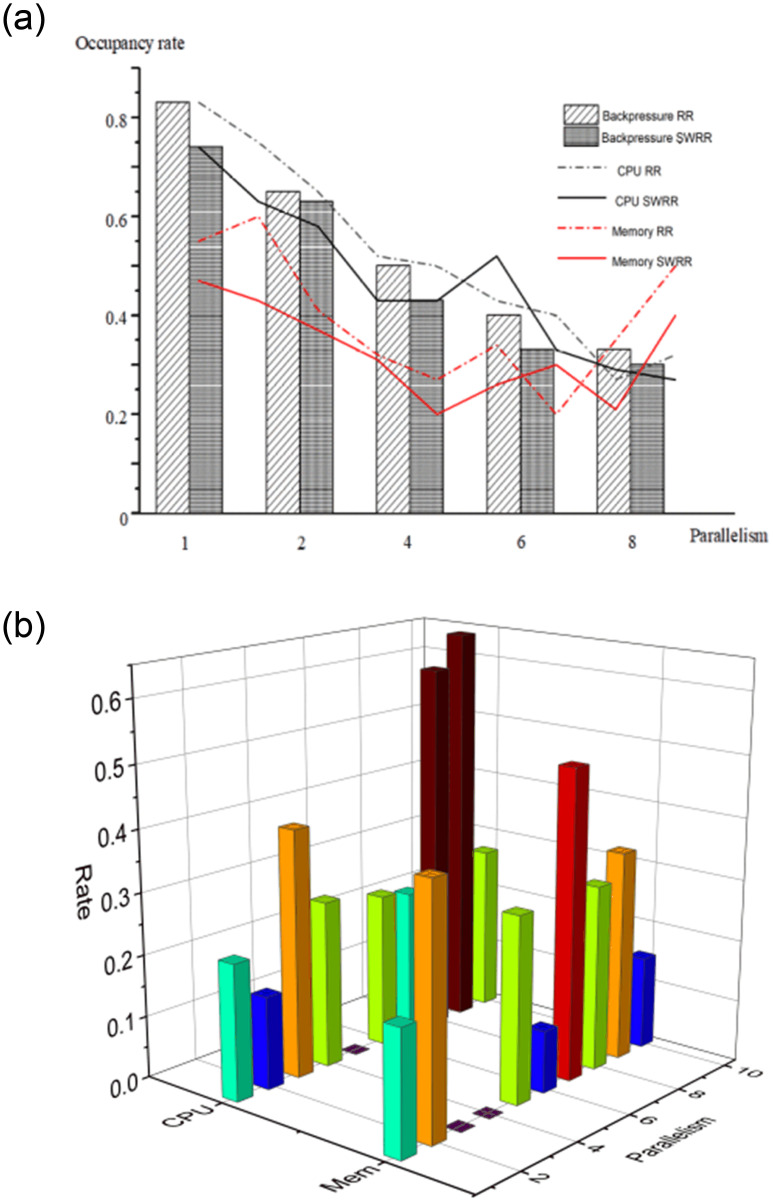
(a) The CPU occupancy rate, memory occupancy rate, and the probability of triggering the BF mechanism of the master node under different parallelisms; (b) CPU and memory occupancy rates under parallelism of 10.

By taking 1.3 × 107 pieces of telemetry data from the 10 kV power scheduling and monitoring the SCADA system for railway power supply as an input data source, the topological parallelism is changed on the condition of having a fixed number of nodes in the cluster. When the parallelism is 1, 2, 4, 6, 8, and 10, the back-pressure mechanism, CPU, and memory occupancy rates are monitored (Fig 6).

Compared with the resource utilization rate of Spark in [17], the CPU usage fluctuates between 75% and 100%, the utilization rate is higher, and the change is smoother, but the power supply monitoring data center needs to reserve resources for scheduling data. Data reservation resources generally require the CPU utilization of the master node to be within 50%, so we adjust the CPU usage by adjusting the parallelism. Fig 6a shows that, with increasing topological parallelism, multiple instances are established for a component to operate in different containers, in which the setting of the task is consistent with that of the executor. According to the equation for L_q_ (Section 4), it can be seen that the intensity of the message service of the system is $\;\rho \;=\;\frac{\lambda }{4\mu }$, and W_s_ and W_q_ are related to parallelism. Therefore, reasonably distributing the parallelism can shorten the waiting time W_q_ of information flow. When the topological parallelism varies from 1 to 8, the CPU occupancy rate of the master node decreases: in particular, when the parallelism is changed from 2 to 4, the CPU occupancy rate varies from 75% to 52% by use of the RR scheduling algorithm; while, when using the SWRR scheduling algorithm, the CPU occupancy rate is changed from 58% to 43%. Moreover, the reduction in memory occupancy rate also reaches about 15%, thus improving the flexibility of the system. Therefore, as the topological parallelism constantly increases, CPU and memory occupancy rates also decrease as expected. When the parallelism is increased to 10, the amplitudes of CPU and memory occupancy rates fluctuate significantly. After monitoring the fluctuation for 10 min (Fig 6b) a 0% CPU occupancy rate is observed and also a low memory occupancy rate is noted, which indicates that the topological processing pauses. Thus, on the premise that resources in a cluster are limited, CPU occupancy rates fluctuate from 65% to 0% while the change in memory occupancy rate does not conform to changes in growing resources when the topological parallelism increases to 10 in blind test conditions. This shows that, under such parallelism, although the back-pressure mechanism is not triggered, resources distributed to the CPU, and memory, are limited and the increasing parallelism means that CPU and memory are subjected to multiple segmentation events. This causes the CPU and memory to suffer high occupancy rates. As a result, topological processing pauses, thus leading to significant fluctuations.

Although the adjustment of topological parallelism has an obvious effect on reducing CPU and memory occupancy rates, memory and CPU occupancy rates fluctuate when topological parallelism is increased to a certain level. This impedes the system in timeous processing of data and nodes may crash.

### Comparison of the occupancy rates of memory of worker nodes in the cluster

**Fig 7:**
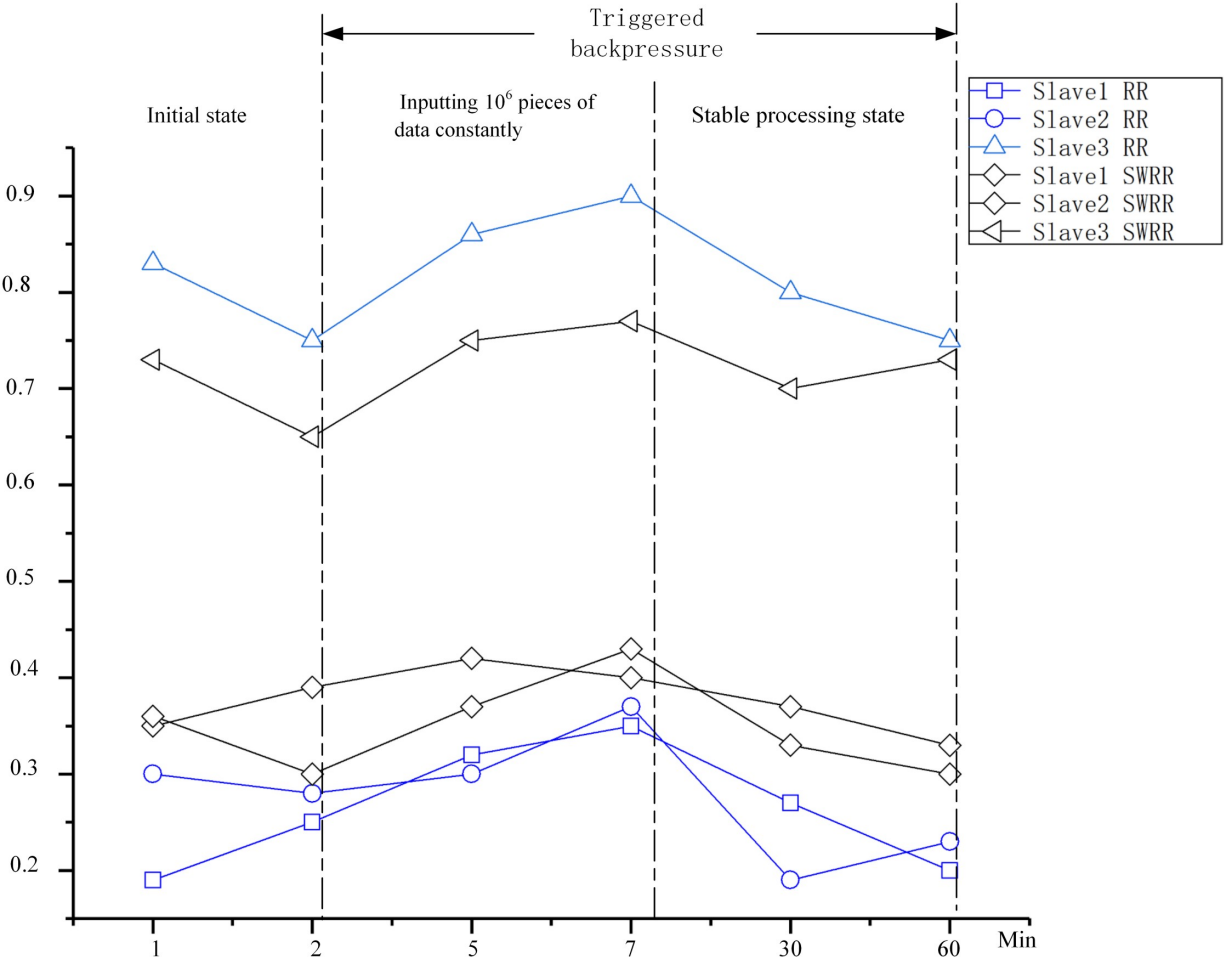
Occupancy rates of memory of worker nodes.

On the condition of having fixed topological parallelism, multiple groups (2.3 × 10^6^, 3.4 × 10^6^, 1.3 × 10^7^) of telemetry data are taken as the input data source. After the topological tasks operated for a certain time in the cluster, the memory occupancy rates of three slave nodes are recorded. By using the two scheduling methods for worker nodes, the occupancy rates of memory of each worker node are compared (Fig 7).

As shown in Fig 7, it can be found that the SWRR algorithm effectively reduces the memory occupancy rate of slave 3 while proportionately increases the memory occupancy rates of nodes 1 and 2, so that each server in the cluster can participate in the process of topological treatment. According to experimental data, it can be seen that the memory occupancy rate of node 3 decreases from 83% to 65%, which implies that scheduling of the cluster is dispersed and servers with favorable performance are not always invoked. The memory occupancy rates of nodes 1 and 2 increase from 20% to about 35% and the utilization rates of the other nodes increase therewith. Data are steadily and proportionately scheduled to other nodes so that the nodes can share the pressure of the cluster, thus improving the processing capacity of the cluster.

When constantly inputting 10^6^ pieces of data, under the RR scheduling algorithm, the memory occupancy rate of slave 1 rises to about 90%. In this case, the response speed of the system decreases significantly. In contrast, the memory occupancy rates of slave 2 and slave 3 are less than 40%, which results in extremely unbalanced loads in the cluster, and the maximum difference in memory occupancy rates reaches 50%. When using the SWRR algorithm, the memory occupancy rate of slave 1 is reduced to 70%, indicating a decrease of 23% compared with that using the RR algorithm. Moreover, the memory occupancy rates of the other nodes increase to 37%.

After triggering the BF mechanism, any data flow can be slowed down and memory occupancy rates then fluctuate within 10%. This indicates that BF-SWRR can eliminate any inflow of a large volume of data within a short time, optimize the scheduling of nodes in the cluster, and avoid discontinuity in topological treatment brought about by unbalanced loads.

### The effect of cluster scheduling mode on the delay time

**Fig 8:**
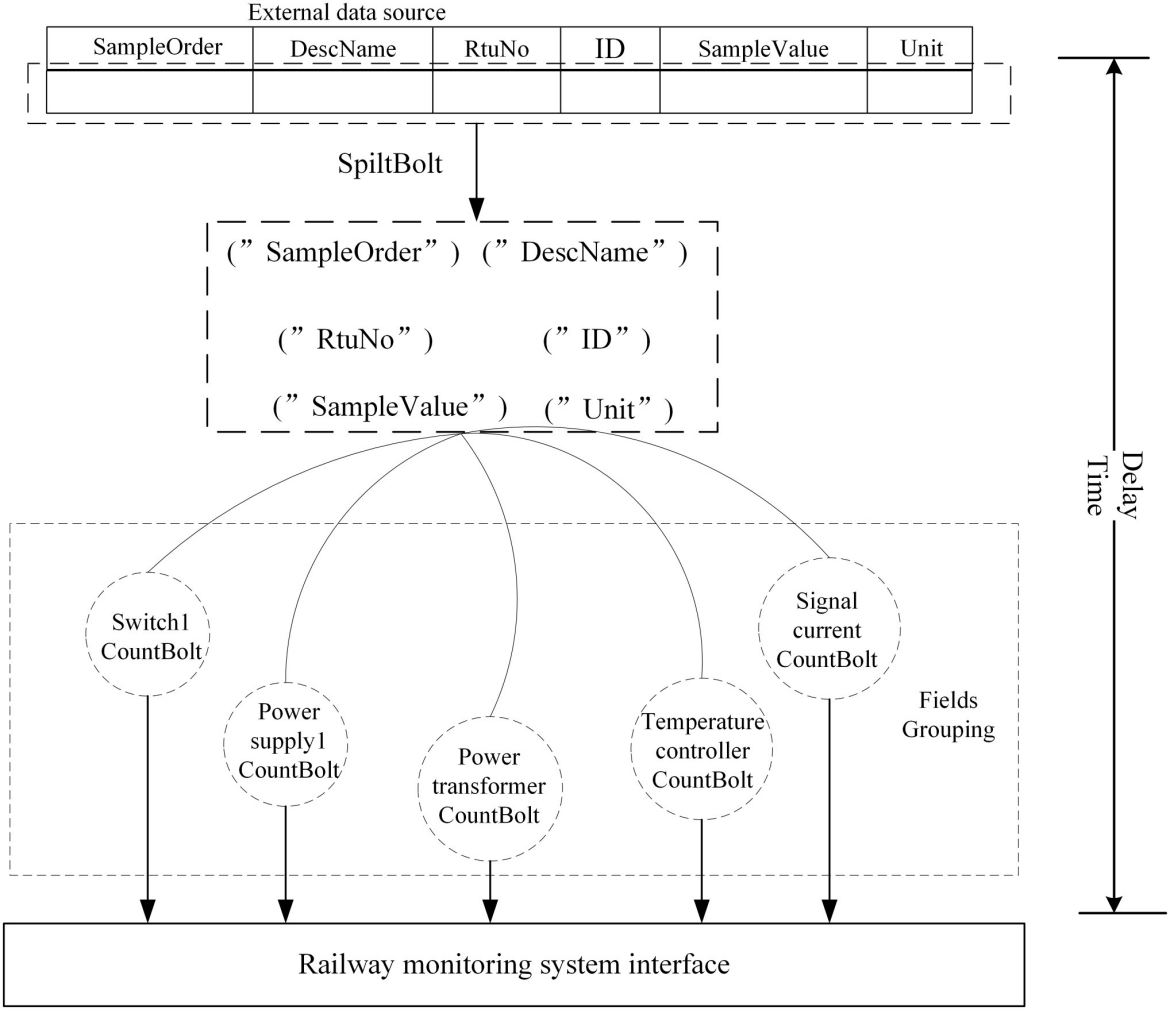
Process of topological treatment.

In engineering practice, data overload occurs, generally accompanying start-up and braking of trains. In this case, various indices (including electric parameters and temperature coefficients) all change and are transmitted to the scheduling center. A crucial monitoring period is thus imminent, therefore, it is necessary to introduce the back-pressure mechanism to the cluster scheduling center. The delay of data processing after slicing is calculated by applying a timestamp during the experiment and the data processing operation is as shown in Fig 8. The method of computing time is summarized as follows: the initial timestamp is marked at the data source Spout while at the end of the experiment, the initial timestamp is subtracted from the time of completion of data processing in CountBolt. By doing so, the delay in data processing can be attained.

**Fig 9:**
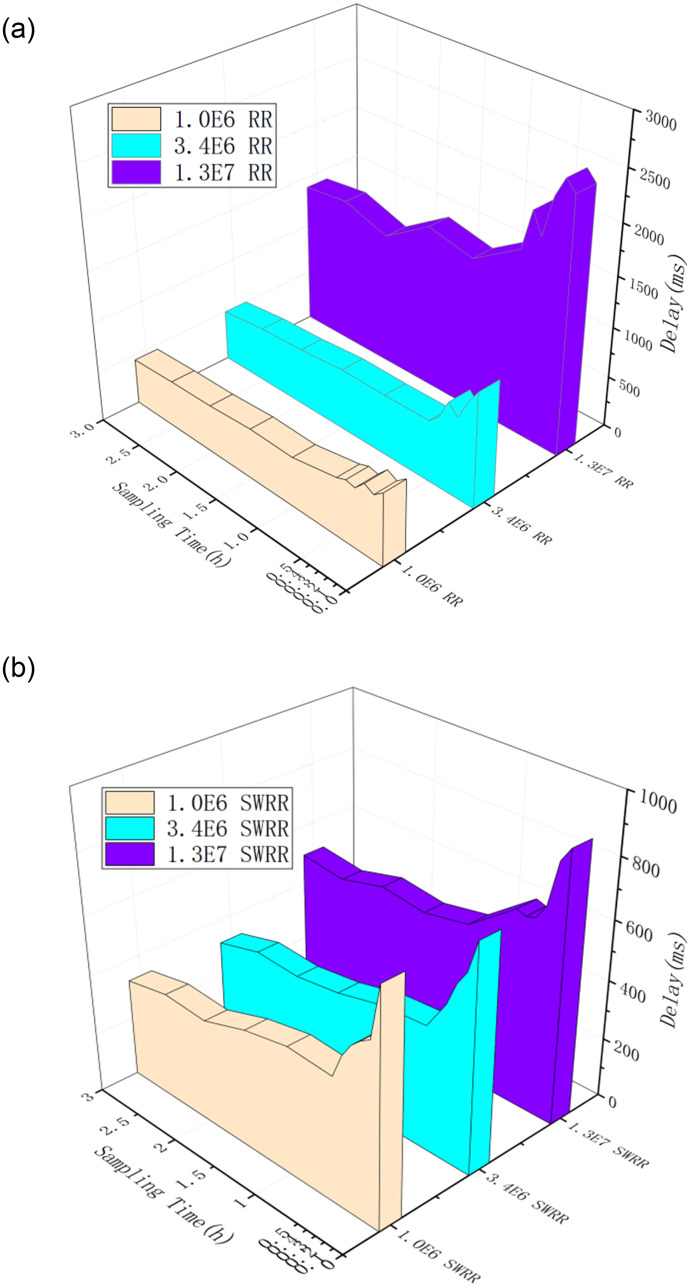
(a) Processing delay of different telemetry data flows under the RR scheduling method; (b). Processing delay of different telemetry data flows under the SWRR scheduling method.

**Fig 10:**
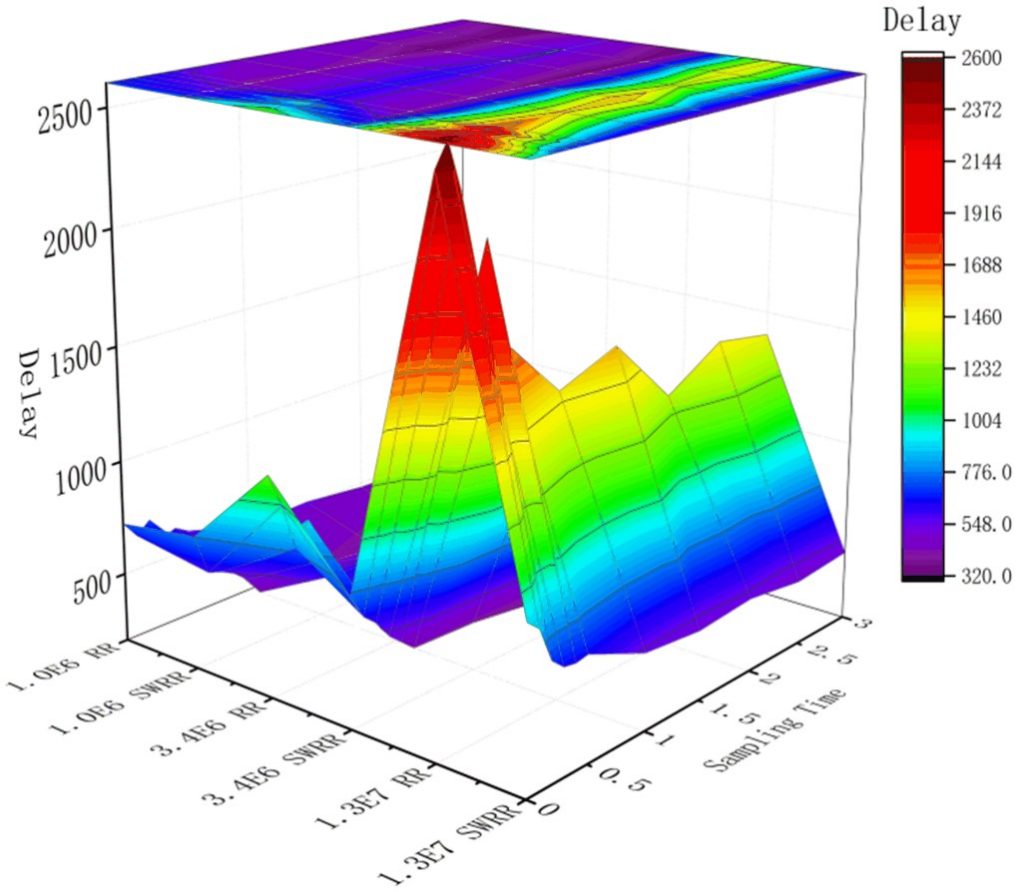
Processing delay of different telemetry data flows.

By taking 1 × 10^6^, 3.4 × 10^6^, and 1.3 × 10^7^ groups of SCADA telemetry data, on condition of having fixed nodes in a cluster and having parallelism of 8, the processing delay of different telemetry data flows is computed, as shown in Figs 9 and 10.

Each node with DBMS-X handles 535 MB, and the delay is about 100 s. The flow calculation model in the present research delays the data at the level of 10^7^ at the level of 100 ms [13], the delay characteristics of the stream computing cluster are better, then the existing algorithm and the improved algorithm of the scheduling center are compared, and it can be concluded Fig 9a and 9b that, under a fixed control scheduling method, the influence of data flow on delay is analyzed from the perspective of different data flows. Within the first 0.5 h, many delays are recorded. It can be found that the delay in data delivery is approximately 1 s (initially) and even reaches 2.5 s for large data. This indicates that the two scheduling methods are both affected by the volume of data in the initial stage; moreover, BF is not triggered timeously, or data flows continue to fluctuate, so it requires some time to stabilize the BF times and data flow.

As shown in Fig 9a, under the RR scheduling method, the telemetry data flows with different orders of magnitude are subject to different delays. The delay of data flow at magnitude 10^6^ is about 500 ms while that of data flow at magnitude 10^7^ (after BF stabilizes) reaches 1800 ms. This implies that the delay is positively correlated with the volume of data flow. Fig 9b shows that, in the initial stage of observation and recording, the delay is generally 20% longer than that from 2 to 3 h, indicating that it is difficult for servers to process the data rapidly in the initial stage when they are uploaded in quantity. When BF is stable after 1 h, delays fall to a normal state. This implies that the BF mechanism is flexible and can be favorably used for dynamic scaling based on current resources.

Fig 10 demonstrates the relationship between delay and different scheduling methods from multiple perspectives (different data flows and scheduling methods). By taking the spectrum as the reference coordinate for a delay, that light with a longer wavelength represents a longer delay. Moreover, trends in data arising from the use of different methods can be favorably reflected. As shown in Fig 10, before 0.5 h, the delay is recorded at intervals of 0.1 h. Through observation, it is found that delays fluctuate, implying that it requires some time to stabilize BF in the invoking stage. Under the RR scheduling method, the delay cannot stabilize to within 1 s in the short-term, and stabilization can take more than 1 h in some cases; in contrast, based on the use of the SWRR scheduling method, the delay can become stable within 0.7s at up to 0.2 h overall, which reduces the pressure caused by data accumulation.

As the system is stable after 0.5 h, the time interval is set to 0.5 h. After conducting multiple observations and recordings, it can be found that the delay in the initial stage of data processing is longer than those in the middle and later stages. In the middle and later stages, owing to the BF mechanism being triggered, data are proportionately distributed to server nodes with different weights so that delay is decreased by 12% to 20%. When the input data size increases from magnitude 10^6^ to 10^7^, the delay remains within 700 ms under SWRR scheduling (a reduction of more than 30% compared with that using the RR scheduling algorithm). This satisfies the requirement for the response of a railway monitoring system to be within 1.5 s. Moreover, it can be found that, even if the data volume increases 10-fold, the streaming computing processing system can still ensure a response within 1 s.

## Conclusion

The present research is aimed at problems incurred such as the processing delay for massive data from a railway power distribution monitoring system, high-occupancy CPU, and unbalanced memory occupancy. When facing the influx of big data, especially data overload instantaneously forms when a high-speed train starts-up, data can first enter Bolt in an orderly manner under the BF mechanism. Moreover, by utilizing a distributed stream computing–topology parallel programming model, the message queues are processed to overcome the drawback of traditional distributed servers that show imbalanced scheduling. Furthermore, a BF-SWRR scheduling model is proposed. The studies show that compared with the RR scheduling mechanism, the use of the BF-SWRR balanced scheduling algorithm can reduce CPU occupancy rates by 15% to 20% and decrease the memory occupancy rate to about 40% by setting a reasonable level of topological parallelism. Additionally, the use of the BF-SWRR scheduling algorithm increases the processing capacity for data overload and shortens the processing time to the order of hundreds of milliseconds, which can improve the dynamic response capability of monitoring massive power distribution telemetry data, and greatly reduce the scheduling processing time of the railway grid.

The research in this paper mainly focuses on the handling of massive structured data, but for sets of massive unstructured data, such as images taken on-site and the video text are taken by track-inspection vehicles, future research is warranted. In future work, we will consider combining neural networks with big data processing and using deep learning-based methods, taking the resource utilization of each node as the learning sample the better to cope with the increasingly large high-speed railway data streams.

## Supporting information

S1 File Original data sources. This is a railway EMU data file.
(ZIP)

S2 File The values used to build Fig 6. (XLS)

S3 File The values used to build Fig 7. (XLSX)

S4 File The values used to build Fig 9. (XLS)

S5 File The values used to build Fig 10. (XLS)

S6 File (ZIP)

S7 File (ZIP)

S8 File (ZIP)

S9 File (ZIP)

S10 File (ZIP)

S11 File (ZIP)
